# Maternal Supplementation with Polyphenols and Omega-3 Fatty Acids during Pregnancy: Prenatal Effects on Fetal Fatty Acid Composition in the Iberian Pig

**DOI:** 10.3390/ani12162140

**Published:** 2022-08-21

**Authors:** Ana Heras-Molina, Rosa Escudero, José L. Pesántez-Pacheco, Consolación García-Contreras, Marta Vázquez-Gómez, Susana Astiz, Cristina Óvilo, Antonio González-Bulnes, Beatriz Isabel

**Affiliations:** 1 CSIC-INIA, Ctra. De La Coruña Km. 7.5, 28040 Madrid, Spain; 2 Faculty of Veterinary Medicine, Universidad Complutense de Madrid, Ciudad Universitaria s/n, 28040 Madrid, Spain; 3 School of Veterinary Medicine and Zootechnics, Faculty of Agricultural Sciences, University of Cuenca, Avda. Doce de Octubre, Cuenca 010220, Ecuador; 4 Nutrition and Obesities: Systemic Approaches Research Unit (NutriOmics), INSERM, Sorbonne Université, 75006 Paris, France; 5 Departamento de Producción y Sanidad Animal, Facultad de Veterinaria, Universidad Cardenal Herrera-CEU, CEU Universities, C/Tirant lo Blanc, 7. Alfara del Patriarca, 46115 Valencia, Spain

**Keywords:** hydroxytyrosol, linseed oil, progeny, swine

## Abstract

**Simple Summary:**

The present study aimed to determine the effects of maternal dietary supplementation combining hydroxytyrosol and n3 polyunsaturated fatty acids (n3-PUFA) from day 35 to day 100 of gestation on the fatty acid (FA) composition of the offspring tissues of the Iberian pig. No effects were found in the plasma FA composition of the dams but higher levels of n3-PUFA were found in the plasma and different tissues (muscle, liver, and brain) of the supplemented fetuses. These findings may have important implications for piglets’ health and may offer guidance for achieving human dietary n3-PUFA recommendations.

**Abstract:**

Intrauterine Growth Restriction (IUGR) is a major problem in pig production and different strategies, mainly maternal supplementation with different agents, are currently being studied. The combination of hydroxytyrosol and n3-PUFA seems to be a promising treatment to counteract IUGR, since the combination may help improve n3-PUFA composition and lower the inflammatory status of IUGR piglets. The aim of the present study is to determine the effects of a maternal supplementation, from day 35 to day 100 of pregnancy, with linseed oil and hydroxytyrosol on the fetal FA composition. The results showed higher n3 levels, including eicosapentaenoic and docosahexaenoic FA in the offspring from treated gilts, which showed lower n6-PUFA/n3-PUFA (n6/n3) ratios. Saturated and monounsaturated fatty acids were also affected by treatment, especially in the muscle and brain. Thus, a maternal supplementation with linseed oil and hydroxytyrosol affected the fetal FA tissue composition, which could have implications in pig production due to the improvement of the piglets’ health status.

## 1. Introduction

Maternal nutrition during pregnancy is critical for the adequate fetal growth and the metabolism of the offspring, with important implications during the offspring’s lifetime [1]. Concerning the importance of pigs in both animal production and translational research, different nutrients have been tested in pregnant sows to improve their offspring’s fetal status and postnatal development [2].

Essential fatty acids (EFA) are studied because some polyunsaturated fatty acids (PUFA) are indispensable for adequate tissue development during fetal stages and, therefore, pregnancy success [3]. In particular, n3-PUFAs are being studied as a supplement for gilts and sows due to their positive effects of improving offspring growth and development [4], metabolism [5], and immune response [6]. Thus, dietary supplementation with n3-PUFAs during pregnancy may be useful for the prevention of events related to intrauterine growth restriction (IUGR), low-birth weight, and mortality [7]. The utilization of n3-PUFA in pig nutrition is also drawing attention due to the resemblance between the FA composition of feed and the tissues in pigs. Thus, aiming to meet the World Health Organization (WHO) recommendations of a n6-PUFA/n3-PUFA (n6/n3) ratio of 4:1 [8], it is possible to implement feeding strategies to increase the n3-PUFA content of pork and thereby increase the n3-PUFA consumption by the human population [9,10]. However, the possible detrimental effects of an excessive intake of PUFA during fetal stages (especially referring to the effects on desaturases and elongases) must be considered in any livestock and human beings prior to any recommendation [11,12], so further research on this subject is needed. 

Furthermore, PUFA are easily oxidized due to their high degree of unsaturation, so their combination with an antioxidant is recommended [13,14]. Hydroxytyrosol is being increasingly studied in pigs because of its high antioxidant capacity and its anti-inflammatory, immune, and metabolism-modulatory properties [15]. Previous results indicated that hydroxytyrosol supplementation may improve the development and metabolism of piglets both pre and postnatally, especially in case of IUGR [16,17,18,19].

The combination of n3-PUFA and hydroxytyrosol has been previously studied in pigs, showing an improvement in the lipidemic and glycemic profiles at the fetal stages, which triggers positive effects on the litter size [20]. Animals obtained from mothers treated with hydroxytyrosol and α-linolenic acid (ALA) showed better growth and lipidemic indexes at postnatal stages, but with controversial results when considering the fatty acid composition of tissues [21]. 

In view of these considerations, the current study aimed to determine the effects of a maternal supplementation with linseed oil and hydroxytyrosol from day 35 to day 100 of pregnancy on the FA composition of the plasma and different tissues of fetuses. 

## 2. Materials and Methods

### 2.1. Ethic Statement

The experiment was performed according to the Spanish Policy for Animal Protection (RD 53/2013), which meets the European Union Directive 2010/63/UE on the protection of research animals. The INIA Committee of Ethics in Animal Research assessed and approved the experimental procedures (report CEEA 2013/036, 19 February 2014). Gilts were housed at INIA animal facilities that are in accordance with local, national, and European requirements for Scientific Procedures Establishments.

### 2.2. Animals and Experimental Procedures

The study involved a total of 131 fetuses obtained from 14 Iberian gilts that were pregnant after cycle synchronization with altrenogest (Regumate^®^, MSD Animal Health, Boxmeer, The Netherlands) and artificial insemination with cooled semen from the same purebred boar. 

During pregnancy, gilts were fed a standard grain-based diet formulated to supply the following mean component values (g/kg of feed): dry matter, 910.7 g/kg; crude protein, 122.8 g/kg; fat, 35.5 g/kg; metabolizable energy, 2910.4 kcal/kg. From the start of the experimental period (insemination day—day 0) to gestational day 35 (day 35), feed allocations were adjusted to fulfill individual daily maintenance requirements based on data from the National Research Council [22]. The most abundant FAs (FA) in the diet were linoleic acid (LA; 41.5 g/100 g total FA), palmitic acid (20.6 g/100 g total FA), and oleic acid (19.3 g/100 g total FA). 

On day 35, all gilts were weighed, and their feed allocation adjusted to fulfill 50% of daily maintenance requirements until delivery. On this same day (35) of pregnancy, the females were pair-matched by body weight to obtain two homogeneous groups of seven gilts per group. Therefore, there were no differences in mean body weight between groups (149.64 ± 7.47 kg vs. 146.57 ± 2.51 kg; *p* = 0.80). Maternal adiposity was also similar (46.67 ± 4.40 vs. 44.57 ± 2.80 mm; *p* = 0.86), which was estimated in terms of backfat depth measured at 4 cm from the midline and transversal to the head of the last rib with a multifrequency linear-array ultrasonographic probe (SV1 Wireless scanner, SonopTek, Beijing, China). One of the groups remained on the same diet (group C), whereas the other group (group T) received an isocaloric diet including 4% of linseed oil and 1.5 mg hydroxytyrosol/kg feed (Supplementary Table S1). The component values of the diet in the treated group were (g/kg feed): dry matter, 910.3 g/kg; crude protein, 123.5 g/kg; fat, 62.3 g/kg; and metabolizable energy, 2909.1 kcal/kg. In the treatment diet, the most prominent FAs were LA (32.3 g/100 g total FA), α-linolenic acid (ALA, 29 g/100 g total FA), and palmitic acid (11.8 g/100 g total FA). The FA methyl esters in the diet were identified by gas chromatography (Hewlett Packard HP-6890, Avondale, PA, USA) with a flame ionization detector and a capillary column (HP-Innowax, 30 m × 0.32 mm i.d. and 0.25 µm polyethylene glycol-film thickness) [23], after extraction and methylation by the one-step procedure proposed by Sukhija and Palmquist [24]. The n6-PUFA intake was 14.78 g/kg and 20.47 g/kg of diets C and T, respectively, and the n3 total intake was 2.37 g/kg and 18.72 g/kg of diets C and T, respectively.

Fetuses were obtained on gestational day 100 (which corresponds approximately to 90% of the 112-days gestation typical of this breed). Of the 131 fetuses obtained, 60 were from control females (group C) and 71 from treated females (group T). On this day, blood samples were drawn from the orbital sinus of the gilts with sterile EDTA 10 mL vacuum tubes (Vacutainer^TM^ Systems Europe, Meylan, France) after 16 h of fastening. Samples were immediately centrifuged at 1500× *g* for 15 min; afterwards, the plasma was separated and biobanked into polypropylene vials at −80 °C until they were assayed for plasma FA composition.

### 2.3. Sampling of Fetuses 

Gilts were euthanized in compliance with RD 53/2013. The content of the uterus was exposed, and fetal sex was determined by visual inspection immediately after recovery. A sample of fetal blood was drawn from the heart and/or umbilical cord using EDTA syringes and processed as previously described for gilts. Then, samples were taken from *longissimus dorsi* muscle, liver, and brain and stored at −20 °C for FA composition analysis. 

### 2.4. Fatty Acid Composition of Plasma and Tissues

Plasma, *longissimus dorsi*, liver, and brain fat were extracted as described by Segura et al. [25] after lyophilization and homogenization, with fat content in each tissue calculated and expressed as a percentage. In the case of the intramuscular tissue, liver, and brain fat, the neutral lipid fraction (triglycerides) and polar lipid fractions (phospholipids) were separated using aminopropyl minicolumns previously activated with 7.5 mL of hexane [26]. The FA composition of all three tissues and plasma were analyzed using gas chromatography [23]. The quantities of individual FAs expressed as g/100 g of total FA content were used to calculate the proportions of saturated FAs (SFA), monounsaturated FAs (MUFA), polyunsaturated FAs (PUFA), and total n3 and n6-PUFA. The n6-PUFA/n3-PUFA (n6/n3) and the MUFA/SFA ratios were also calculated. The unsaturation index (UI) was determined as follows: 1[%monoenoics] + 2[%dienoics] + 3[%trienoics] + 4[%tetraenoics] + 5[%pentaenoics] + 6[%hexaenoics] [27,28].

Assessment of the activity of the stearoyl-CoA desaturase enzyme 1 was performed using the ratio C18:1/C18:0 (DI; [29]), while the total Stearoyl-CoA desaturase and Palmitoyl-CoA desaturase activity (D9) was assessed with the formula (C16:1n7 + C18:1n9)/(C16:1n9 + C18:1n7 + C18:0 + C16:0). The enzyme activities for n6 and n3 FAs were calculated by the ratio C20:4n6/C18:2n6 (DN6) and C20:5n3/C18:3n3 (DN3), respectively.

### 2.5. Statistical Analysis 

Data were analyzed using SPSS 25.0 (IBM Corp., Armonk, NY, USA). Verification of normal distribution was completed with a Kolmogorov–Smirnov test. The homogeneity of variances was studied with an F-test. Data from gilts were analyzed using the Student’s t test. To analyze fetal data, effects of diet (control vs. treatment) and sex (female vs. male) on developmental traits, adiposity, FA composition, oxidative stress, and metabolic status were assessed using three-factor ANOVA for diet, sex, and litter size. Due to the bias between treatment and litter size previously described [20], litter size was also considered an effect. Animals were grouped by their litter sizes after determining the mean number of fetuses per litter (9.25 ± 1.42 piglets per sow) and defining small litters as those with ≤9 piglets and large litters as those with >9 piglets.

Relationships between maternal plasma and fetal plasma FAs were determined using Pearson correlation procedure. Statistical significance was considered when *p* < 0.05, whereas a trend was considered when 0.1 > *p >* 0.05.

## 3. Results

### 3.1. Fat Content of the Different Tissues

No differences between the treatments were found in the total fat of the *longissimus dorsi* (9.25 ± 0.14% in group C vs. 9.30 ± 0.15% in group T), livers (15.18 ± 0.72% in group C vs. 14.30 ± 0.59% in group T), or brains (37.83 ± 0.90% vs. 37.44 ± 0.81%) of the fetuses. 

### 3.2. Fatty Acid Composition of the Plasma of the Gilts and Fetuses

No significant differences were found in the fatty acid (FA) compositions of the plasma of the control and treated gilts at day 100 of gestation (Table 1). However, the maternal treatment affected the plasma FA proportions of the fetuses (Supplementary Table S2). Regarding SFA, the group T fetuses had higher levels of myristic (C14:0) and palmitic acids (C16:0). The stearic acid (C18:0) showed a triple interaction between treatment*sex*litter size (*p* < 0.05). Hence, group C males had higher C18:0 concentrations than group C females in small litters, while group T females had higher values than group C males in large litters; females had greater concentrations than males in both treatment groups. Individual MUFA such as cis-7 hexadecenoic acid (C16:1n9) were affected by the litter size (*p* < 0.05, higher concentration in small than large litters), while other MUFA such as palmitoleic (C16:1n7) and vaccenic acids (C18:1n7) were affected by sex (higher concentrations in males than females and *p* < 0.05 for C16:1n9 and *p* < 0.01 for C18:1n7).

**Figure 1:**
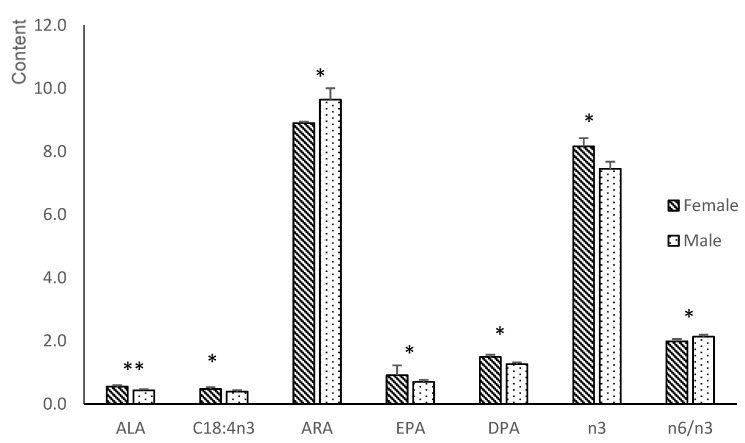
Significant differences in the n3 and n6-PUFA series in plasma samples between female and male fetuses obtained at 100 days of gestation born from control gilts or from gilts treated with hydroxytyrosol and linseed oil from day 35 to day 100 of gestation. ALA = α-linolenic acid; ARA = arachidonic acid; EPA = eicosapentaenoic acid; DPA = docosapentaenoic acid; n3 = total sum of n3-Polyunsaturated fatty acid; n6 = total sum of n6-Polyunsaturated fatty acid. * *p* < 0.05; ** *p* < 0.01.

The supplementation affected the LA levels in the PUFA content; therefore, fetuses in group T had higher concentrations than fetuses in group C (*p* < 0.05). Sex also had an important effect on the FA composition of the offspring’s plasma (Figure 1). Thus, males had a higher n6/n3 ratio than females (*p* < 0.05) due to their higher concentration of ARA and lower levels of ALA, EPA, and docosapentaenoic acid (C22:5n3; DPA; *p* < 0.05 for all except ALA; *p* < 0.01). On the other hand, dihommo-gamma-linolenic acid (C20:3n6) showed a treatment*sex interaction (*p* < 0.05). Males from Group T had a higher concentration of C20:3n6 than those in Group C (*p* < 0.05), with no differences in females.

Differences were also observed between small and large litters, especially in the PUFA group. Small litters had lower levels of n3-PUFA but a higher percentage of n6-PUFA when compared to large litters; therefore, small litters also showed a higher n6/n3 ratio (*p* < 0.05 for n6-PUFA difference and *p* < 0.001 for n3-PUFA and n6/n3 PUFA ratios). Again, those dissimilarities were mainly due to different concentrations of ALA and EPA, which were higher in large litters than in small litters (*p* < 0.01,), and ARA (*p* < 0.001; being higher in small litters than in large litters). Adrenic acid (C22:4n6) showed a treatment*sex interaction (*p* < 0.05). Among the group C fetuses, small litters had a greater percentage of this FA than large litters (*p* < 0.001), but small and large litters from group T had similar values.

### 3.3. Fatty Acid Composition of the Longissimus Dorsi Muscle of the Fetuses

#### 3.3.1. Neutral Fraction

**Figure 2:**
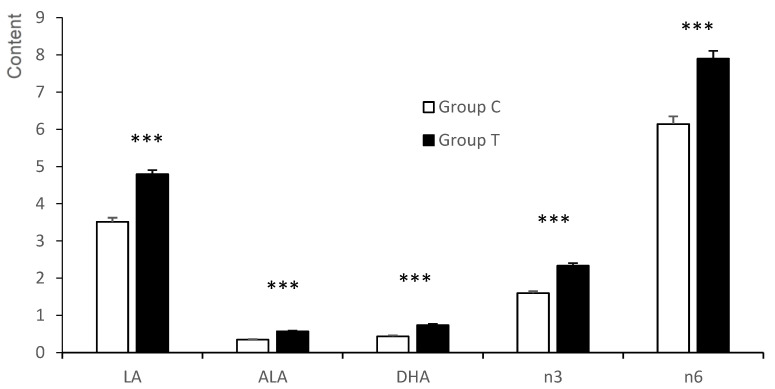
Significant differences in the n3 and n6-PUFA series of the neutral fraction of the longissimus dorsi muscle between fetuses obtained at 100 days of gestation born from control gilts (group C) or from gilts treated with hydroxytyrosol and linseed oil from day 35 to day 100 of gestation (group T). LA = linoleic acid; ALA = α-linolenic acid; DHA = docosahexaenoic acid; n3 = total sum of n3-Polyunsaturated fatty acid; n6 = total sum of n6-Polyunsaturated fatty acid. *** *p* < 0.001.

The total SFA was similar between the groups in the neutral fraction of the *longissimus dorsi* muscle (LD; Figure 2 and Supplementary Table S3), but there were differences when assessing SFA individually. Concretely, the content of palmitic acid (C16:0) was higher in the fetuses of group C than in group T (*p* < 0.05), while both heptadecanoic acid (C17:0) and C18:0 showed a treatment*litter-size interaction (*p* < 0.05 for both). In both FAs, group C showed a greater concentration in small than in large litters (*p* < 0.05), whilst there was a greater concentration of C17:0 in the large litters than in the small ones in group T. Content of MUFA was higher in fetuses of group C when compared to fetuses of group T (*p* < 0.001). Concretely, C16:1n9, oleic acid (C18:1n9) and C18:1n7 for MUFA were higher in the group C fetuses (*p* < 0.001 for C18:1n7 and *p* < 0.05 for the others). On the other hand, heptadecenoic acid (C17:1) showed a treatment*litter size interaction (*p* < 0.05), with large litters having greater values than small litters in group T (*p* < 0.05) and similar concentrations in group C. A treatment*litter size interaction was found in the DI (*p* < 0.05). Thus, there were differences between group C and group T animals from large litters (*p* < 0.01, greater values in the group C fetuses), but no differences were found in small litters. 

Total PUFA, as well as n3 and n6-PUFA and UI, were again higher in the fetuses of group T than in group C (*p* < 0.001 for all). Differences among the n3-PUFA were mostly driven by ALA (*p* < 0.001), DPA (*p* < 0.01), and DHA (*p* < 0.001), all of them having greater content in group T than in group C. Furthermore, the LA, gamma linolenic acid (C18:3n6), and C20:3n6 were also in higher concentrations in the fetuses of group T (*p* < 0.001 for all). The n6/n3 ratio suffered a treatment*litter size interaction (*p* < 0.05), with fetuses in group C having similar values, but there were higher values for the fetuses in the small litters of group T than in the small ones of group C (*p* < 0.01). The activity of DN3 was higher in the fetuses of group C than in group T (*p* < 0.05).

#### 3.3.2. Polar Fraction 

The total SFA showed no differences in the polar fraction of the LD (Supplementary Table S4). However, C17:0 showed a treatment*litter size interaction (*p* < 0.001): within the small litters, only a trend wherein the group C fetuses had a higher C17:0 concentration was found (*p* = 0.065); whereas within the large litters, group T had higher concentrations than the group C fetuses (*p* < 0.001). Furthermore, C18:0 was in a greater proportion in group C than in the group T fetuses (*p* < 0.05) and in large than small litters (*p* < 0.01). 

Regarding MUFA content, eicosenic acid (C20:1n9) was higher in the group C than in the group T fetuses (*p* < 0.05). The litter size affected C16:1n9, which was in greater proportions in the small litters than in the large ones (*p* < 0.001). In addition, C18:1n7 showed a treatment*litter size interaction (*p* < 0.05), with similar results between the large and small litters in group C, but with a higher concentration in the large litters than in the small ones within group T (*p* < 0.01). There was also a treatment*litter size interaction in D9 (*p* < 0.05). There was a numerically higher value in the large litters than in the small ones in group C, whereas small litters had a numerically higher D9 activity than large ones within group T. 

**Figure 3:**
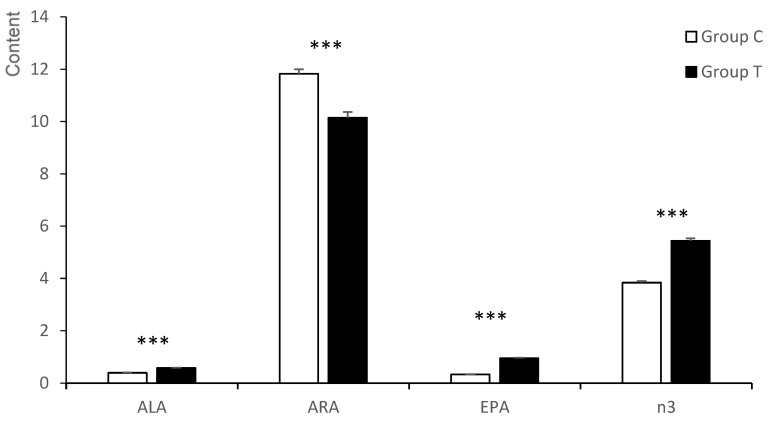
Significant differences in the n3 and n6-PUFA series of the polar fraction of the *longissimus dorsi* muscle between fetuses obtained at 100 days of gestation born from control gilts (group C) or from gilts treated with hydroxytyrosol and linseed oil from day 35 to day 100 of gestation (group T). ALA = α-linolenic acid; ARA = arachidonic acid; EPA = eicosapentaenoic acid; n3 = total sum of n3-Polyunsaturated fatty acid. *** *p* < 0.001.

The content of n3-PUFA was higher in the group T than in the group C fetuses (*p* < 0.001; Figure 3). Individual n3-PUFA ALA, EPA, and DPA were in greater concentrations in the group T fetuses (*p* < 0.001), while the DHA showed a significant treatment*litter size interaction (*p* < 0.05): it had similar values within litter sizes in the group C fetuses, but in group T animals it was in a higher concentration in the large litters than in the small ones (*p* < 0.01). The total n-6 showed no significant difference, as LA and C20:3n6 were higher in the group T fetuses and ARA was higher in the group C ones (*p* < 0.001). The n6/n3 ratio showed a treatment*litter size interaction (*p* < 0.05). Thus, group C fetuses showed similar values between litter sizes, while group T fetuses showed greater values in small than in large litters (*p* < 0.05). The DN3 activity was higher in the group T fetuses, and the DN6 activity was greater in the group C than in the group T animals (*p* < 0.001 for both).

### 3.4. Fatty Acid Composition of the Liver of the Fetuses

#### 3.4.1. Neutral Fraction 

**Figure 4:**
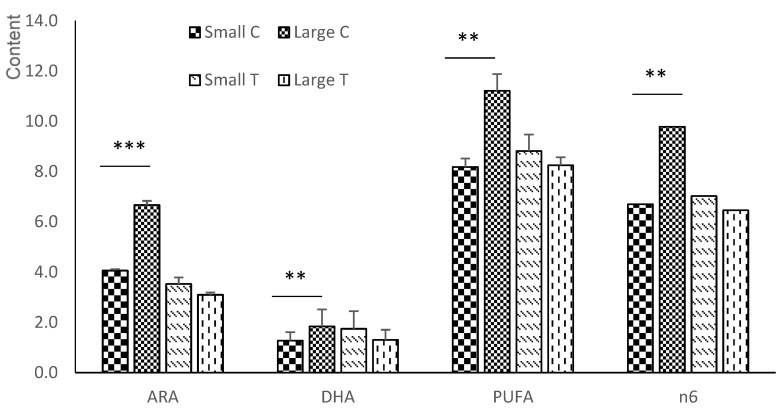
Treatment*litter size interactions in the n3 and n6-PUFA series of the liver between fetuses obtained at 100 days of gestation born from control gilts (group C) or from gilts treated with hydroxytyrosol and linseed oil from day 35 to day 100 of gestation (group T). ARA = arachidonic acid; DHA = docosahexaenoic acid; PUFA = sum of polyunsaturated fatty acids; n6 = sum of n6-PUFA. ** *p* < 0.01; *** *p* < 0.001.

In the neutral fraction of the liver (Figure 4 and Supplementary Table S5), the total SFA was affected by a treatment*litter size interaction (*p* < 0.05), with small litters having higher a concentration of SFA than in the large litters within the group C fetuses (*p* < 0.001), whereas within the group T fetuses, this outcome was similar between the small and large litters. At the individual FA level, only C17:0 showed differences between treatment groups (*p* < 0.05; higher levels in group T fetuses) and litter sizes (*p* < 0.05; higher concentration in small litters). 

On the other hand, MUFA were in a higher proportion in the large than in the small litters (*p* < 0.05). When analyzed individually, C17:1 was in a greater concentration in the group T than the group C fetuses, whereas C18:1n7 was higher in the group C than group T fetuses (*p* < 0.01 for both). The MUFA/SFA ratio and D9 activity were more affected by the litter size, having greater values in large than small litters (*p* < 0.01 and *p* < 0.05, respectively).

Regarding PUFA, there was a treatment*litter size interaction (*p* < 0.01). Within the group C fetuses, large litters had higher values than small ones (*p* < 0.001); within the group T fetuses, small litters had similar values to large ones. When studying n6-PUFA, a treatment*litter size interaction was also observed, with the differences between treatment groups being greater in large litters (*p* < 0.001, greater values in the group C fetuses), whereas similar values in the group C and group T fetuses were observed in small litters. This interaction was mainly due to the similar treatment*litter size interaction found in ARA (*p* < 0.001). The total n3-PUFA did not show any significant difference, but DHA showed a treatment*litter size interaction (*p* < 0.01), with the group C fetuses from large litters having greater concentrations than the group C fetuses from small ones (*p* < 0.001) and, in group T, small litters had higher values than large ones (*p* = 0.055). On the other hand, C22:5n3 was higher in group T than in group C animals (*p* < 0.001).

#### 3.4.2. Polar Fraction 

**Figure 5:**
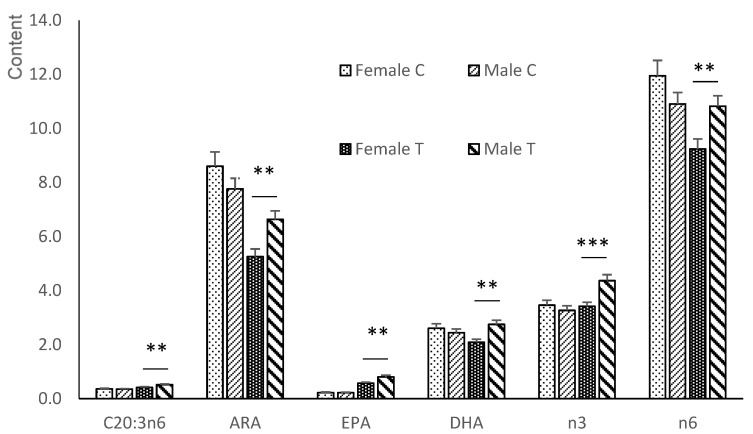
Treatment*sex interactions of the polar fraction of the liver in fetuses between fetuses obtained at 100 days of gestation born from control gilts (group C) or from gilts treated with hydroxytyrosol and linseed oil from day 35 to day 100 of gestation (group T). ARA = arachidonic acid; EPA = eicosapentaenoic acid; DHA = docosahexaenoic acid; n3 = sum of n3-PUFA; n6 = sum of n6-PUFA. ** *p* < 0.01; *** *p* < 0.001.

In the polar fraction of the liver (Figure 5 and Supplementary Table S6), group T fetuses and small litters had a higher SFA concentration than group C fetuses and large litters (*p* < 0.05 and *p* < 0.01, respectively). Among individual FA, C14:0, C16:0 and C18:0 showed different treatment*sex interactions (*p* < 0.05 for all). Assessment of C14:0 showed similar concentrations in males from group C and T, but females in group T had higher C14:0 values than females in group C (*p* < 0.05). Content of C16:0 was similar between both sexes in group C, while females had higher levels than males in group T (*p* < 0.05). Finally, C18:0 was higher in group C females than counterpart males and in group T males than counterpart females, although significant differences were only found in group T (*p* < 0.05). Furthermore, C14:0 was in greater content in small than large litters (*p* < 0.05), while C17:0 showed a treatment*litter size (greater differences between small and large litters in group C whereas within group T it had similar concentration; *p* < 0.05).

Treatment*sex interactions were observed in total MUFA, C16:1n7 and C18:1n9, as well as D9 and DI (group C: higher values in males than females; group T: higher values in females than males, *p* < 0.05 for both). On the other hand, C18:1n7 was in higher concentration in group C than group T animals (*p* < 0.001). Litter size also affected individual MUFA such as C18:1n7 (*p* < 0.05, higher concentration in large litters) and C17:1, affected by a treatment*litter interaction (*p* < 0.05). Concretely, in group C there were higher values in small than large litters (*p* < 0.05) while in group T there was a similar concentration in both litter sizes).

Total, n3- and n6-PUFA, UI and DN6 were affected by the same treatment*sex interaction (group C: females had similar values to males; group T: males had significantly greater values than females; *p* < 0.01). This same interaction was also found within individual n3 such as EPA (*p* < 0.05), DHA (*p* < 0.01) and within n6 FA such as C20:3n6 and ARA (*p* < 0.01 for both). In addition, a treatment*litter size interaction was found in total PUFA, n6 and DN6, consisting of large litters having greater values than small ones within the group T fetuses while within group C animals the values were more similar (*p* < 0.05 for PUFA, *p* < 0.01 for n6-PUFA and *p* < 0.001 for DN6). Finally, the n6/n3 ratio was higher in group C than the group T fetuses (*p* < 0.001), whereas DN3 activity was higher in group T than group C animals (*p* < 0.001).

### 3.5. Fatty Acid Composition of the Brain

#### 3.5.1. Neutral Fraction 

In the neutral fraction of the brain, the SFA was affected by sex (*p* < 0.01; greater values in females than males). Similarly, C17:0 and C18:0 were in higher concentrations in females when compared to males (*p* < 0.05 for both). 

The total MUFA was similar among groups. However, at the individual FA level, various treatment*litter size interactions were found. Thus, C16:1n9 was higher in the group C than in the group T fetuses in small litters and in group T than in the group C fetuses in large ones, although no significant differences were achieved between groups (*p* < 0.05). A similar treatment*litter size interaction was found in the case of C16:1n7 (*p* < 0.05), with greater differences between treatment groups within small litters (*p* < 0.05), whereas no significant differences were found in the large litters. Regarding C18:1n9, the fetuses in group T had greater concentrations than in group C when considering small litters (*p* < 0.05), whereas no differences were found in large litters (*p* < 0.01).

Regarding PUFA, C22:4n6 was affected by sex (*p* < 0.01, higher values in males than females). The content of DHA showed a treatment*sex interaction: females had lower concentrations in group C than in the group T fetuses, whereas males had greater DHA concentrations within group C than in group T (*p* < 0.05). The n6/n3 ratio showed a treatment*sex*litter size interaction (*p* < 0.05). In this case, in the small litters, both males and females in group T had greater values than in group C, while females from group C showed significantly higher values than females from group T in large litters; no differences were found when comparing males. 

#### 3.5.2. Polar Fraction

**Figure 6:**
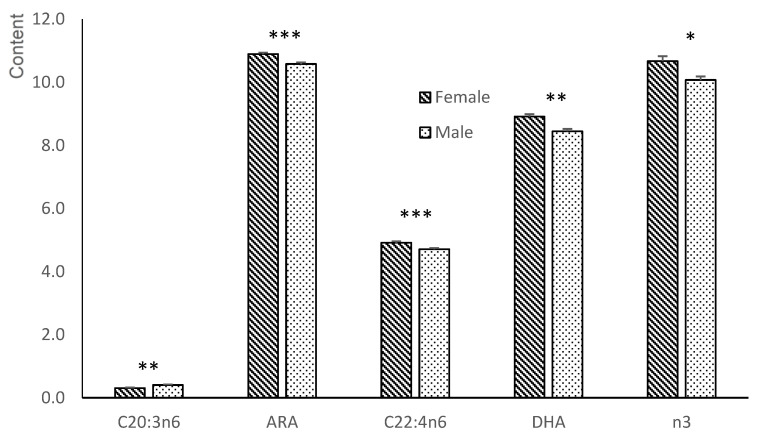
Significant differences in the n3 and n6-PUFA series of the polar fraction of the brain between female and male fetuses obtained at 100 days of gestation born from control gilts or from gilts treated with hydroxytyrosol and linseed oil from day 35 to day 100 of gestation. ARA = arachidonic acid; DHA = docosahexaenoic acid; n3 = sum of n3-PUFA. * *p* < 0.05; ** *p* < 0.01; *** *p* < 0.001.

The total SFA and MUFA/SFA were affected by a treatment*litter size interaction (Figure 6 and Supplementary Table S7). On the one hand, the SFA showed higher levels in the group C fetuses from small litters than in the group C fetuses from large ones (*p* < 0.01), while the group T fetuses showed similar values among litter sizes (*p* < 0.01). This interaction was also found for C16:0 (*p* < 0.001). On the other hand, the MUFA/SFA ratio was higher in group T animals within small litters (*p* < 0.05) and similar between treatments of large litters (*p* < 0.05). the total MUFA was affected by sex (*p* < 0.001; greater values in males than females). 

The total n6-PUFA showed a treatment*sex*litter size interaction: within small litters, similar values between sexes were found in the group C fetuses, but females had greater n6-PUFA concentrations in group T; within large litters, similar values between sexes were found in group T, but females had a greater concentration than males in group C (*p* < 0.05). On the other hand, the n3-PUFA concentration was affected by sex, as it was in higher concentrations in females than males (*p* < 0.05). This difference was mainly due to the higher percentage of DHA in females than males (*p* < 0.01), as this FA also affected by litter size (*p* < 0.01; higher concentration in the large litters than in the small ones). The activity of DN6 was affected by both sex (higher in males than females; *p* < 0.001) and litter size (*p* < 0.01; higher values in small than large litters).

## 4. Discussion

The results of the present study indicate that maternal supplementation with n3-PUFA in the form of linseed oil and hydroxytyrosol during gestation affects the plasma and tissue FA composition of piglets. It must be considered that the maternal treatment diet was a fat-enriched feed (35 g/kg and 62 g/kg for C and T groups respectively), so higher concentrations of n6-PUFA and total n-3 PUFA were administered in group T. Thus, to overcome such a difference, the FA composition of the tissues was expressed in relative terms (g/100 g total FA) instead of absolute ones.

The assessment of the plasma FA composition of the gilts showed no significant differences between groups with or without supplementation, contrary to previous research [30]. However, there are several differences between our study and previous studies that may be the cause of such dissimilarities. First, the animals had different ages and were from different breeds (with presumptive differences in uterine and placental development [31]). Second, they had different gestational ages (which may affect the maternal plasma FA composition, because the transplacental transport of lipids increases with fetal demands [32,33]). Such differences may also be responsible for the lack of correlation between the plasma FA composition of the mothers and fetuses. However, we cannot leave aside other plausible explanations related to the lipogenic activity de novo that occurs in the adipose tissue and the liver of the pig fetuses [34,35] or the high utilization of FAs in the synthesis of new tissues during the fetal stages [36]. 

In our study, in contrast to the gilts, fetuses in groups T and C showed differences in the FA composition of all tissues (plasma, muscle, liver, and brain). The group T fetuses had higher levels of n3-PUFA in all tissues, with higher concentrations of ALA and elongated FAs such as EPA and DHA. Thus, even though the conversion rate from ALA to EPA and DHA is only about 5% [37], the supplementation with ALA is confirmed as an effective way to increase the concentrations of other n3-PUFA in Iberian pigs, in agreement with previous research in other breeds [38,39]. 

The differences found in the PUFA compositions among the organs after maternal supplementation may be derived from the differential tissue expression of the elongase-2 enzyme, which is vital for the final conversion of EPA to DHA [40]. Although organs such as the brain or liver also showed an increase in n3-PUFA concentration in group T, muscle was the tissue with the more prominent difference between treatments, in accordance with previous studies [41]. Litter size also had important implications in the n3-PUFA concentrations, as it was highly related to the treatment group. Previous studies with this population have reported a trend for larger litters in group T than in group C (*p* = 0.075) [20], which could explain this result. Sex also had important effects on the n3-PUFA accumulation. Previous studies have shown that maternal supplementation has different implications for males and females in swine [16,41], with offspring showing different metabolic adaptations depending on their sex [42], so the diet restriction given in the present experiment could have reinforced the differences between males and females regarding n3-PUFA accumulation. There are sex-specific differences in essential n-3 essential fatty acid metabolism [43]. The latter authors observed that sex hormones may influence the enzymatic synthesis of long-chain polyunsaturated fatty acids. On the other hand, Childs [44] reported that women have greater increases in their EPA status after ALA supplementation than men, and a growing body of animal model research identifies the mechanism by which sex hormones such as estrogen and progesterone interact with the synthesis of EPA and DHA. However, further research is required in pigs to understand the effect of increasing ALA levels during pregnancy, which in our study were different depending on the tissue.

Interestingly, n6-PUFAs were also affected by the treatment, as they were significantly higher in the neutral fraction of the longissimus dorsi muscles of group T. This outcome may be explained by the hydroxytyrosol supplementation, which is in agreement with previous research in Iberian pigs at prenatal stages [17]. Supporting the data of Garcia-Contreras et al. [17], and the study performed using oleuropeins by Rey et al. [45], our results indicate a protective effect of the polyphenol over PUFAs. The different effect of hydroxytyrosol at the muscle and at the organs may be related to the diet restriction given in the experiment. In case of compromised fetal nutrition, as in the present study, the growth of essential organs (the brain and liver) is protected at the expense of other organs [30,46] and mainly at the expense of muscle development [47,48,49]. Hence, the effects of hydroxytyrosol supplementation are more evident in the muscle than in other organs such as the liver or the brain, as its development is more challenged. 

In the current study, the concentrations of SFA and MUFA were also affected by the treatment, although differently in each tissue. Fetuses can synthesize SFA and MUFA [50,51], so it is possible that maternal supplementation affected the metabolic pathways of FA accretion in different tissues. This outcome has been previously seen in piglets from gilts supplemented during gestation and lactation with PUFA at postnatal stages [51]. In this regard, dietary PUFA can affect the lipid metabolism of all kinds of FAs, possibly due to their relationship with intracellular SFA, the higher affinity for carrier proteins such as the FA binding protein, or the changes induced in the cellular membrane with implications in the response to insulin [52,53]. 

The FA composition of different tissues at postnatal stages was previously studied with a similar design [21]. The results indicated that, in the *longissimus dorsi* fat, a higher percentage of PUFA in group T and higher n6/n3 ratio in group C were maintained at 60 days-old. However, the higher abundance of n3-PUFA was lost at this age. Later, at 180 days-old, there were no differences in n3 or total PUFA content in the intramuscular fat, but a lower n6-PUFA concentration was found in group T. Conversely, differences were maintained in the polar fraction of the liver over time. At 60 days-old, the higher concentration of n3-PUFA found in the current study at the fetal stages was maintained; however, at 180 days-old, both n3 and n6-PUFA concentrations were lower in group T. Therefore, joining both current and such previous studies, the activity of enzymes implicated in the elongation and desaturation of PUFAs may be affected by maternal supplementation and such an effect may affect the pig during its lifetime through processes of prenatal programming. However, further studies would be necessary to confirm such a hypothesis. 

## 5. Conclusions

The present study shows that maternal supplementation with n3-PUFA and hydroxytyrosol modifies the FA composition of the different tissues of porcine offspring at the fetal stages. These effects are strongly modulated by sex- and litter size. In brief, the supplementation increases the PUFA proportion, especially of n3-PUFA. Furthermore, SFA and MUFA were also affected by the treatment. Thus, these results aid the understanding of the effects of maternal supplementation on the progeny at prenatal stages and could have important consequences for pig production. From a practical point of view, the improvement of the PUFA profile, and above all n3-PUFA, can have positive implications for the health status of piglets and, afterwards, for the meat characteristics. However, further studies from the postnatal stages until the sacrifice for pork production would be needed to fully ascertain the effects of the maternal supplementation given herein on the meat characteristics.

## Figures and Tables

**Table 1:** Fatty acid composition of the plasma at day 100 of gestation of control gilts (group C) or gilts treated with hydroxytyrosol and n3-PUFA from day 35 to day 100 of gestation (group T).

Fatty Acid (g/100 g Total FA)	Group C	Group T	Pooled S.E.M.	*p*-Value
C14:0	2.73	3.20	0.54	0.682
C16:0	18.98	20.45	1.83	0.705
C16:1n9	2.20	3.50	0.43	0.137
C16:1n7	2.99	4.34	0.86	0.999
C17:0	1.99	2.06	0.68	0.710
C17:1	2.43	1.59	0.71	0.805
C18:0	14.50	16.19	1.88	0.670
C18:1n9	20.14	14.37	2.23	0.207
C18:1n7	2.77	2.44	0.23	0.490
C18:2n6 (LA)	12.99	11.49	2.48	0.777
C18:3n6	1.32	1.28	0.35	0.620
C18:3n3 (ALA)	1.67	2.25	0.37	0.805
C18:4n3	1.12	1.50	0.25	0.209
C20:1n9	2.45	2.82	0.55	0.747
C20:3n6	1.00	1.15	0.20	0.730
C20:4n6 (ARA)	3.25	3.10	0.42	0.865
C20:5n3 (EPA)	1.22	2.07	0.34	0.227
C22:4n6	2.76	2.54	0.43	0.815
C22:5n3 (DPA)	2.20	2.26	0.42	0.945
C22:6n3 (DHA)	1.30	1.38	0.16	0.806
SFA	38.20	41.91	3.02	0.561
MUFA	32.98	29.07	1.60	0.236
PUFA	28.83	29.03	2.54	0.969
UI	1.24	1.25	0.08	0.987
MUFA/SFA	0.86	0.69	0.08	0.302
n3	7.51	9.47	1.20	0.437
n6	21.32	19.56	2.27	0.716
n6/n3	2.84	2.07	0.75	0.165

LA = linoleic acid; ALA = α-linolenic acid; ARA = arachidonic acid; EPA = eicosapentaenoic acid; DPA = docosapentaenoic acid; DHA = docosahexaenoic acid; SFA = sum of saturated fatty acids; MUFA = sum of monounsaturated fatty acids; PUFA = sum of polyunsaturated fatty acids; UI = unsaturation index; n3 = sum of n3-PUFA; n6 = sum of n6-PUFA.

## Data Availability

Data are contained within the article.

## Supplementary Materials

The following are available online at https://www.mdpi.com/article/10.3390/ani12162140/s1, Table S1. Estimated analysis (g/kg), ingredient (g/kg; left table), and fatty acid composition (g/100 g total fatty acids) of the experimental diets of Control gilts (group C) and treated with hydroxytyrosol and n3-PUFA (Treated; group T); Table S2A. Fatty acid composition of the plasma of fetuses born from control gilts (group C) or gilts treated with hydroxytyrosol and n3-PUFA from day 35 to day 100 of gestation (group T); Table S2B. Fatty acid composition of the plasma of fetuses born from control gilts (group C) or gilts treated with hydroxytyrosol and n3-PUFA from day 35 to day 100 of gestation (group T) and from small (≤9 piglets) or large (>9 piglets) litters; Table S3A. Fatty acid composition of the neutral fraction of the longissimus dorsi intramuscular fat of fetuses born from control gilts (group C) or gilts treated with hydroxytyrosol and n3-PUFA from day 35 to day 100 of gestation (group T); Table S3B. Fatty acid composition of the neutral fraction of the longissimus dorsi intramuscular fat of fetuses born from control gilts (group C) or gilts treated with hydroxytyrosol and n3-PUFA from day 35 to day 100 of gestation (group T) and from small (≤9 piglets) or large (>9 piglets) litters; Table S4A. Fatty acid composition of the polar fraction of the longissimus dorsi intramuscular fat of fetuses born from control gilts (group C) or gilts treated with hydroxytyrosol and n3-PUFA from day 35 to day 100 of gestation (group T); Table S4B. Fatty acid composition of the polar fraction of the longissimus dorsi intramuscular fat of fetuses born from control gilts (group C) or gilts treated with hydroxytyrosol and n3-PUFA from day 35 to day100 of gestation (group T) and from small (≤9 piglets) or large (>9 piglets) litters; Table S5A. Fatty acid composition of the neutral fraction of the liver of fetuses born from control gilts (group C) or gilts treated with hydroxytyrosol and n3-PUFA from day 35 to day 100 of gestation (group T); Table S5B. Fatty acid composition of the neutral fraction of the liver of fetuses born from control gilts (group C) or gilts treated with hydroxytyrosol and n3-PUFA from day 35 to day 100 of gestation (group T) and from small (≤9 piglets) or large (>9 piglets) litters; Table S6A. Fatty acid composition of the polar fraction of the liver of fetuses born from control gilts (group C) or gilts treated with hydroxytyrosol and n3-PUFA from day 35 to day 100 of gestation (group T); Table S6B. Fatty acid composition of the polar fraction of the liver of fetuses born from control gilts (group C) or gilts treated with hydroxytyrosol and n3-PUFA from day 35 to day 100 of gestation (group T) and from small (≤9 piglets) or large (>9 piglets) litters; Table S7A. Fatty acid composition of the neutral fraction of the brain of fetuses born from control gilts (group C) or gilts treated with hydroxytyrosol and n3-PUFA from day 35 to day 100 of gestation (group T); Table S7B. Fatty acid composition of the neutral fraction of the brain of fetuses born from control gilts (group C) or gilts treated with hydroxytyrosol and n3-PUFA from day 35 to day 100 of gestation (group T) and from small (≤9 piglets) or large (>9 piglets) litters; Table S8A. Fatty acid composition of the polar fraction of the brain of fetuses born from control gilts (group C) or gilts treated with hydroxytyrosol and n3-PUFA from day 35 to day 100 of gestation (group T); Table S8B. Fatty acid composition of the polar fraction of the brain of fetuses born from control gilts (group C) or gilts treated with hydroxytyrosol and n3-PUFA from day 35 to day 100 of gestation (group T) and from small (≤9 piglets) or large (>9 piglets) litters
